# ProCoS: Degradation-Robust Manipulation Localization for Special Equipment Inspection Images

**DOI:** 10.3390/s26123647

**Published:** 2026-06-08

**Authors:** Guilong Chen, Guixiong Liu, Weili Luo

**Affiliations:** 1 School of Mechanical and Automotive Engineering, South China University of Technology, Guangzhou 510640, China; 202510180479@mail.scut.edu.cn; 2 Zhuhai Anyes Technology Co., Ltd., Zhuhai 519080, China; 13798981945@139.com

**Keywords:** special equipment inspection, image manipulation localization, degradation robustness, degradation-resilient synergistic learning, prototypical contrastive learning

## Abstract

Manipulation localization in Special Equipment Inspection (SEI) images is crucial for trustworthy industrial supervision. However, existing image manipulation localization models struggle to handle the compound degradation disturbances commonly encountered in SEI scenarios. To address this issue, we propose a Prototypical Contrastive-Synergistic Network (ProCoS). The proposed model enhances consistent discrimination between clean and degraded observations through the Degradation-Resilient Synergistic Learning architecture, while introducing a Prototypical Contrastive Learning mechanism to improve stable representation under compound degradation. Furthermore, we construct SEI-Asym, a manipulation localization dataset for SEI scenarios, and establish a compound degradation evaluation protocol based on orthogonal experimental design. Experimental results show that ProCoS achieves an F1 of 0.6531, an AUC of 0.9670, and an IoU of 0.5485 on SEI-Asym, and reduces $\bar{\Delta F1}$ and ${\left(\Delta F1\right)}_{\max}$ under compound degradation to 0.0873 and 0.1966, respectively. The proposed model provides an effective technical pathway for trustworthy perception, anomaly discrimination, and industrial supervision based on SEI images.

## 1. Introduction

With the continuous advancement of smart cities and digital industrial supervision, Special Equipment Inspection (SEI) is shifting from traditional manual checking to digital forensics and remote supervision [1]. Inspection images are not only evidence carriers but also media for visual perception and status recording, and their authenticity and integrity directly affect process traceability and the credibility of regulatory conclusions. As image editing tools [2] and generative models [3] rapidly evolve, manipulation has become easier and more concealed, creating practical challenges for trustworthy forensics in inspection workflows. Unlike general visual manipulation, manipulation in SEI images has strong semantic constraints specific to the business context and is typically centered on key evidence in inspection procedures. Typical cases include inspector face replacement [4,5] and equipment nameplate splicing [6,7]. Such manipulations often keep global scene naturalness while introducing substantive local semantic deception. Therefore, image-level authenticity classification alone is insufficient for trustworthy supervision tasks such as manual review, and pixel-level manipulation localization in SEI scenarios is both forensically meaningful and industrially valuable [8].

Recent manipulation localization models have achieved clear progress in architecture design, clue fusion, and localization accuracy [9,10,11]. However, most studies are based on public general-purpose datasets and model manipulations under relatively ideal observation conditions [12]. As a result, models often rely excessively on fragile cues such as clear boundaries and local high-frequency anomalies, making them less suitable for compound degradation common in SEI images. In addition, manipulated regions and degraded background regions are more likely to overlap in feature space, which increases both false positives and false negatives. Existing evaluation protocols are also mostly based on single degradation settings [13] and cannot fully reflect the continuous compound degradation in real SEI operating conditions.

**Figure 1:**
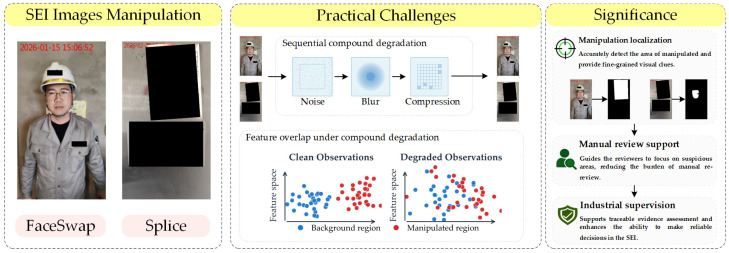
Illustration of challenges and significance of SEI image manipulation localization.

As shown in Figure 1, we aimed to build a pixel-level SEI manipulation localization model under compound degradation that maintains consistent discrimination and stable class separation, providing interpretable evidence for manipulation localization, manual review support, and industrial supervision. To this end, we propose ProCoS, which improves robustness through cross-observation synergistic learning and stable representation modeling: the former reduces reliance on incidental texture shifts and fragile edge artifacts, while the latter enhances intraclass compactness and interclass separability between manipulated and background regions. We also construct the SEI-Asym dataset and a compound degradation evaluation protocol based on orthogonal design [14] for systematic validation.

- We propose Degradation-Resilient Synergistic Learning (DRSL), which explicitly incorporates degraded observations into SEI manipulation localization training and aligns predictions between clean and degraded observations to stabilize cross-observation responses.
- We design a Prototypical Contrastive Learning (PCL) mechanism that dynamically maintains manipulation/background prototypes and enforces explicit intraclass pulling and interclass pushing on student features to improve stable class structure modeling under degradation.
- We construct SEI-Asym, a manipulation localization dataset for SEI scenarios, and establish a compound degradation evaluation protocol based on orthogonal experimental design. The proposed model achieves state-of-the-art performance on SEI-Asym and also demonstrates strong generalization across datasets. Under compound degradation, ProCoS reduces $\bar{\Delta F1}$ and ${\left(\Delta F1\right)}_{\max}$ to 0.0873 and 0.1966, respectively.

## 2. Related Work

Existing studies on image manipulation localization can be reviewed from three aspects: model architectures, robustness under degradation, and manipulation localization dataset and evaluation for SEI.

### 2.1. Image Manipulation Localization

Manipulation localization methods based on deep learning have evolved from Convolutional Neural Network (CNN) architectures to Transformer architectures and then to hybrid designs. CNN-based models mainly focus on local anomaly modeling. For example, ManTra-Net [15] and MVSS-Net [16] extract local anomalous features based on VGG [17] and ResNet [18,19] backbones, respectively, and MVSS-Net further enhances edge and region localization via multiscale supervision. Transformer-based models emphasize global dependency modeling. For instance, TruFor [20] and ProFact [21] adopt a SegFormer style [22] encoder–decoder paradigm, where TruFor focuses on cross-modal fusion and anomaly map generation, while ProFact emphasizes coarse-to-fine feedback refinement. IML-ViT [23] is the first to introduce Vision Transformer [24] into manipulation localization, integrating broader contextual information. Furthermore, hybrid architectures have become an important direction. For example, TransForensics [25] and ObjectFormer [26] combine local convolutional representations with long-range relation modeling, while Mesorch [27] extracts features in parallel using CNN and Transformer branches to jointly model artifacts at the micro level and semantics at the macro level.

From the perspective of discriminative clues, existing models also show a trend from RGB-only modeling to fusion of multiple forensic cues. BusterNet [28] and PSCC-Net [29] mainly model visual anomalies or spatial correlations from RGB images. In contrast, CAT-Net [30] and Mesorch [27] introduce frequency domain information via Discrete Cosine Transform (DCT) [31]; TruFor [20] uses features derived from DnCNN [32,33]; and MMFusion [34] further combines filters such as SRM [35], Bayar [36], and DnCNN [32] to exploit multimodal complementarity.

Overall, manipulation localization has advanced in network design, cue utilization, and accuracy, with growing integration of global context, local details, and multiple forensic cues. However, most models are still optimized for conventional conditions and pay limited attention to stable discrimination under complex degradation, making them hard to transfer directly to the SEI setting studied here.

### 2.2. Robustness of Image Manipulation Localization

The robustness of image manipulation localization refers to stable localization across different application scenarios, including generalization across datasets, robustness under different degradations, and discriminative stability induced by feature space constraints. For generalization robustness, some studies reduce reliance on salient edge artifacts or specific local textures and learn more transferable forgery representations. For example, PIM [37] explicitly models pixel inconsistency to improve generalization on disturbed images and unseen datasets, while GIFL [38] extracts universal features from original image content to alleviate dependence on specific manipulation traces. For degradation robustness, CRL-Net [39] improves localization on JPEG-compressed images through a compression-aware design. For discriminative robustness, methods such as MPC [40] and NCL [41] enhance intraclass compactness and interclass separability through pixel-level contrastive learning, region relation modeling, and constrained feature space optimization, thereby improving robustness to postprocessing operations or unseen samples. Overall, existing studies have extended robust manipulation localization from regular condition accuracy to cross-data generalization, degradation adaptation, and stable discriminative representation learning, providing important inspiration for this work.

In summary, robust manipulation localization research has begun to shift from purely improving localization performance to pursuing stable discrimination under degraded conditions. These advances provide important inspiration for studying manipulation localization under compound degradation. Nevertheless, although some robust manipulation localization studies have considered more stable manipulation representations, key limitations remain: there is generally a lack of explicit constraints on consistent discrimination between clean and degraded observations, and there is also insufficient dedicated handling of feature overlap between manipulated regions and degraded background regions.

### 2.3. Manipulation Localization Dataset and Evaluation for SEI

Special equipment and industrial vision inspection scenarios often face complex imaging conditions, such as weld defect assessment involving multiple experts [42], multiscale detection of complex X-ray defects [43], and key point localization of bolts in complex backgrounds [44]. In these scenarios, industrial vision tasks typically require the development of scene-specific data and evaluation frameworks. Existing image manipulation localization datasets can be mainly divided into general local manipulation datasets and face forgery datasets. In general local manipulation datasets, samples are usually constructed by using general segmentation algorithms such as SAM [45] to obtain target objects and masks, followed by postprocessing methods such as Poisson blending [46] to weaken abrupt pasted boundaries. In contrast, face forgery datasets usually rely on face swapping algorithms such as FaceFusion [47] to generate forged results, and further use face segmentation methods such as SegFace [48] to produce manipulation region annotations.

Table 1 compares representative datasets with the proposed SEI-Asym dataset in terms of application scenario. As shown in Table 1, although existing public datasets support research on general region manipulation and face forgery, they are still insufficient for SEI scenarios. Their manipulated objects, motivations, and business semantic constraints differ clearly from those of SEI images. Moreover, SEI images often contain unmanipulated objects with the same semantics as the manipulated ones, such as other inspector faces or different equipment nameplates, requiring methods to localize the truly manipulated regions under the same semantic interference. Therefore, SEI-Asym is specifically constructed for evidence-related object manipulation and the same semantic interference in SEI scenarios, rather than serving as a simple supplement to existing public datasets.

In addition, benchmarks such as IMDL-BenCo [13] offer relatively systematic evaluation pipelines and test robustness to single degradations, including JPEG compression, Gaussian blur, and Gaussian noise. However, most evaluations remain based on a single degradation source and therefore cannot fully reflect the continuous compound degradation characteristics caused by image acquisition, transmission, and postprocessing in SEI scenarios.

Overall, prior work has advanced model design, fusion of multiple forensic cues, robustness research, and benchmark construction. Yet three bottlenecks remain: limited consistency modeling between clean and degraded observations, insufficient constraints for stable class structure, and dataset/evaluation settings that are still misaligned with real operating conditions.

## 3. Method

**Figure 2:**
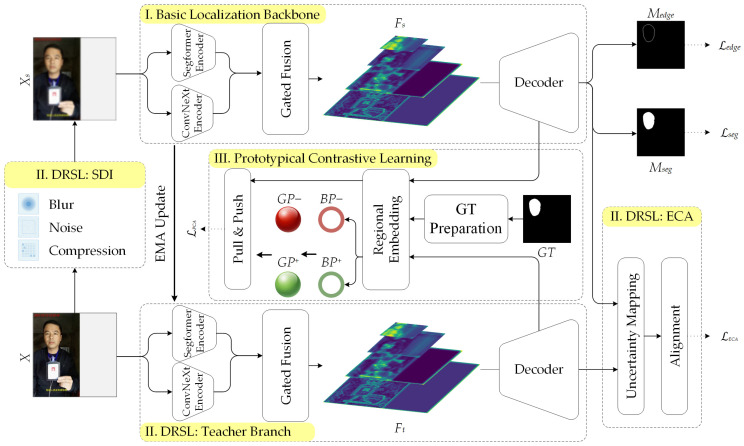
Overview of ProCoS. ProCoS is a unified, robust localization framework for SEI images under complex degradation, consisting of a basic localization backbone, DRSL, and PCL. The backbone learns complementary global semantic and local detail features for pixel-level localization. DRSL enforces consistency between clean and degraded observations, while PCL uses dynamic prototypes to improve intraclass compactness and interclass separability, yielding accurate localization and more stable responses under degradation.

To tackle response drift and feature overlap between manipulation and background in degraded SEI images, we propose ProCoS, as shown in Figure 2. ProCoS jointly optimizes localization accuracy, cross-observation consistency, and representation stability, delivering accurate localization in normal conditions and more robust responses under complex degradation.

### 3.1. Basic Localization Backbone

**Figure 3:**
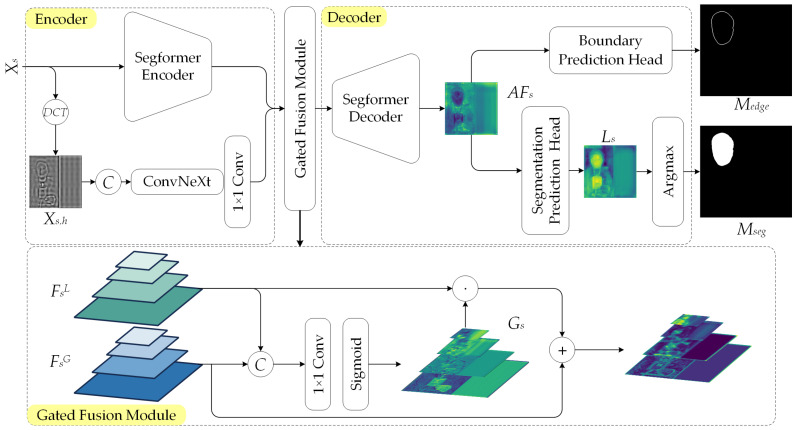
Architecture of the basic localization backbone. The backbone consists of an encoder, a gated fusion module, and a decoder. The encoder extracts global semantic and local detail features, the gated fusion module adaptively combines them at multiple scales, and the decoder aggregates fused features to predict both manipulation regions and boundaries.

The architecture of the basic localization backbone is illustrated in Figure 3. Serving as the representation core of ProCoS, it integrates global semantics and local details to produce discriminative multiscale features for manipulation region prediction and edge prediction.

#### 3.1.1. Encoder

In the backbone, the student branch is the main prediction branch. During inference, it takes clean images and outputs manipulation localization results. During training, it takes degraded images $X_{s}$ and is additionally constrained by teacher–student consistency and prototype contrastive supervision. Given input $X_{s}$, the encoder includes two complementary paths. The global semantic path uses a SegFormer encoder to extract multiscale semantic features ${\left\{F_{s,i}^{G}\right\}}_{i=1}^{4}$ for long-range context and overall modeling of manipulated structures. The local detail path first applies DCT to extract high-frequency components $X_{s,h}$, which are concatenated with $X_{s}$ along channels and fed into ConvNeXt to extract multiscale local detail features ${\left\{F_{s,i}^{L}\right\}}_{i=1}^{4}$. To ensure aligned fusion, local detail features are further projected by convolution so that both paths are aligned in channel dimension.

#### 3.1.2. Gated Fusion Module

After obtaining encoder features from the two paths, ProCoS applies a gated fusion module at each scale to adaptively integrate global and local cues. For the *i*-th scale, a gate map $G_{s,i}$ is generated from channel concatenated features:

(1) $$G_{s,i}=\sigma ({\mathrm{Conv}}_{1\times 1}([F_{s,i}^{G},F_{s,i}^{L}]))$$

where σ(⋅) is the Sigmoid activation, ${\mathrm{Conv}}_{1\times 1}(\cdot )$ is convolution, and [⋅] denotes channel concatenation.

The fused feature $F_{s,i}$ at the *i*-th scale is then computed by position-wise modulation:

(2) $$F_{s,i}=F_{s,i}^{G}+G_{s,i}⊙F_{s,i}^{L}$$

where ⊙ denotes element-wise multiplication.

This module dynamically balances global consistency and local sensitivity. Positions relying more on structure retain stronger global semantics, while positions with local forensic cues (e.g., edge residues or texture disturbances) receive stronger local detail contributions.

#### 3.1.3. Decoder

The SegFormer decoder produces aggregated features AFs∈RH4×W4×256. On this basis, ProCoS uses two task branches: a segmentation prediction head and an edge prediction head. The former outputs a binary prediction $L_{s}\in {\mathbb{R}}^{H\times W\times 2}$ to generate the predicted manipulation mask $M_{seg}\in {\mathbb{R}}^{H\times W\times 1}$, focusing on the overall coverage of manipulated regions; the latter outputs a predicted edge mask $M_{edge}\in {\mathbb{R}}^{H\times W\times 1}$ to strengthen modeling of manipulation boundaries, further improving edge delineation accuracy while balancing region completeness and edge localization quality.

Accordingly, let the ground truth segmentation mask and ground truth edge mask be $Y_{seg}$ and $Y_{edge}$. The basic localization backbone is jointly supervised by segmentation loss $L_{seg}\;$ and edge loss $L_{edge}$:

(3) $$L_{seg}\;=CE(L_{s},Y_{seg}),L_{edge}=BCE(M_{edge},Y_{edge})$$

where CE(⋅,⋅) denotes the pixel-wise cross-entropy (CE) loss, and BCE(⋅,⋅) denotes the binary cross-entropy (BCE) loss [57].

Combining the consistency constraint $L_{ECA}$ in Section 3.2 and the prototype constraint $L_{PCL}$ in Section 3.3, the final optimization objective is as follows:

(4) $$L=L_{seg}+\alpha \cdot L_{edge}+\beta \cdot L_{ECA}+\gamma \cdot L_{PCL}$$

where α,β,γ are weighting coefficients.

Overall, the backbone establishes a unified feature basis for subsequent cross-observation consistency learning and stable class structure modeling.

### 3.2. Degradation-Resilient Synergistic Learning

DRSL is designed to suppress localization drift caused by compound degradation. It explicitly integrates consistency learning between clean and degraded observations through three steps: construction of degraded samples, teacher–student dual-branch collaboration, and output space consistency constraint.

#### 3.2.1. Stochastic Degradation Injection

SDI generates degraded samples online during training. For clean input X, degraded input $X_{s}$ is defined as follows:

(5) $$X_{s}=T(X;k,\sigma ,q)$$

where T randomly samples from Gaussian blur, Gaussian noise, and JPEG compression; corresponding parameters include blur kernel size, noise standard deviation, and JPEG quality k,σ,q.

#### 3.2.2. Teacher–Student Dual Branch Independent Updating

In DRSL, the student branch takes degraded images and is updated by standard backpropagation. The teacher branch takes clean images and provides a stable reference prediction. Teacher parameters are updated by Exponential Moving Average (EMA) [58]:

(6) $$\theta_{t}\leftarrow m_{1}\cdot \theta_{t}+(1-m_{1})\cdot \theta_{s}$$

where $m_{1}\in [0,1]$ is the momentum coefficient.

This design provides temporally smoothed supervisory signals, helping the student maintain stable discrimination under degraded inputs.

#### 3.2.3. Entropy-Modulated Consistency Alignment

**Figure 4:**
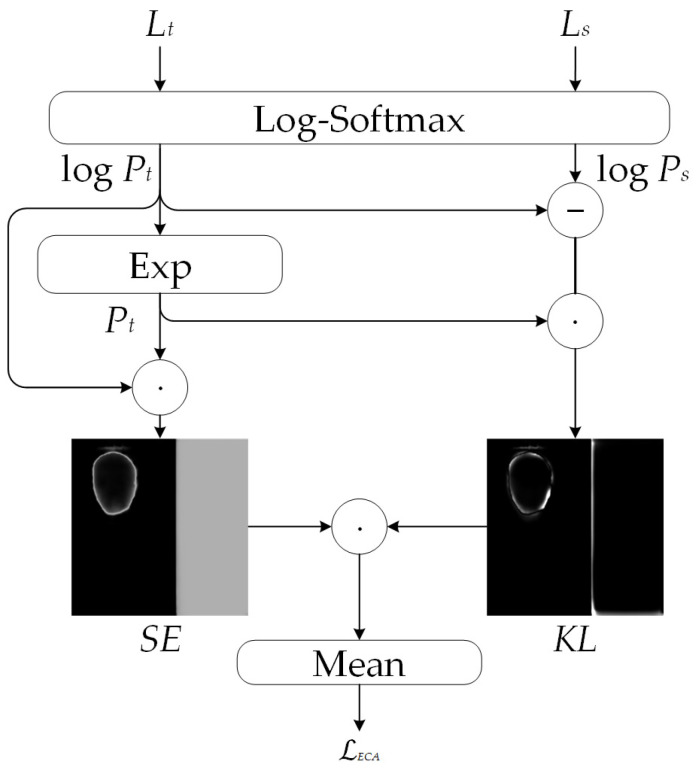
Diagrams of the Entropy-modulated Consistency Alignment (ECA) module. ECA aligns predictions from clean and degraded observations with entropy-aware weighting. High-confidence regions are emphasized to enforce reliable cross-observation consistency, while uncertain regions are given lower weights to reduce noise transfer.

The detailed design of ECA is shown in Figure 4. ECA is introduced to improve cross-observation consistency between clean and degraded inputs without overfitting to unreliable predictions. By modulating alignment strength with entropy, it strengthens supervision on confident regions and suppresses ambiguous responses, thereby improving robustness under compound degradation.

First, student and teacher logits are mapped into log-probability space using Log-Softmax:

(7) $$\log P_{s}=\mathrm{Log}\text{-}\mathrm{Softmax}(L_{s}),\;\log P_{t}=\mathrm{Log}\text{-}\mathrm{Softmax}(L_{t}),\;P_{t}=\exp(\log P_{t})\;$$

where $L_{t}$ is the binary prediction output of the teacher branch.

ECA uses two maps to model cross-observation consistency. The Shannon entropy map SE [59] characterizes teacher uncertainty:

(8) $$SE=-\sum_{c\in \{0,1\}}P_{t}^{c}⊙\log P_{t}^{c}$$

where c∈{0,1} denotes the class channel, and $P_{t}^{c},\;\log P_{t}^{c}$ denote the teacher prediction probability and its log-probability for class c.

The KL divergence map KL [60] measures teacher–student discrepancy under degradation:

(9) $$KL=\sum_{c\in \{0,1\}}P_{t}^{c}⊙\left(\log P_{t}^{c}-\log P_{s}^{c}\right)$$

Finally, entropy-aware spatial modulation is applied to the KL divergence to form the following consistency term:

(10) $$L_{ECA}=-\frac{1}{N}\sum_{n=1}^{N}SE_{n}\cdot KL_{n}$$

where $SE_{n},KL_{n}$ denote entropy and KL values at each pixel, and N is the number of pixels.

Thus, ECA adaptively controls consistency strength according to teacher reliability and improves cross-observation alignment robustness.

### 3.3. Prototypical Contrastive Learning

**Figure 5:**
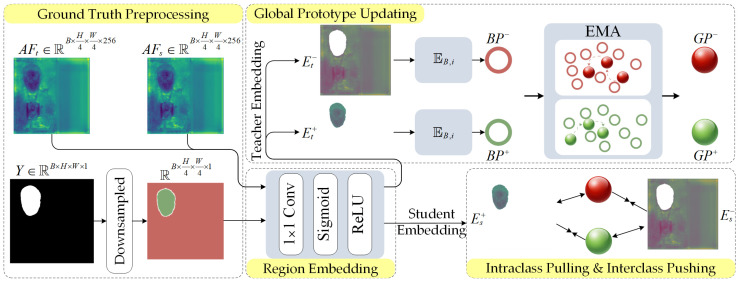
Diagrams of Prototypical Contrastive Learning (PCL). First, ground truth segmentation mask (GT) maps are preprocessed to define manipulation and background regions. Second, region embeddings are extracted from intermediate features. Third, global class prototypes are updated with batch statistics. Fourth, contrastive optimization pulls features toward same-class prototypes and pushes them away from opposite-class prototypes.

As shown in Figure 5, PCL stabilizes intermediate feature structure under complex degradation, reduces overlap between manipulation and background features, and improves class discrimination for more robust localization.

#### 3.3.1. Ground Truth Preprocessing

Since prototype construction uses batch-level statistics, batch dimension is explicitly included. Let the teacher and student aggregated features be $AF_{t},AF_{s}\in {\mathbb{R}}^{B\times \frac{H}{4}\times \frac{W}{4}\times 256}$, and GT be $Y_{seg}\in {\mathbb{R}}^{B\times H\times W\times 1}$. GT are downsampled by nearest neighbor interpolation [61] to match feature resolution, then split into manipulation region and background region masks.

#### 3.3.2. Region Embedding

Prototype contrastive constraints require a unified feature space. Therefore, aggregated features over each region from both teacher and student branches are projected into a shared embedding space. Specifically, region masks are first used to filter aggregated features from the teacher and student branches, yielding aggregated features $AF_{t}^{\pm },AF_{s}^{\pm }$ for the manipulation region and the background region. These features are then mapped by the region embedding function to obtain region embeddings for both branches:

(11) $$\left\{\begin{array}{c} E_{t}^{\pm }=\mathrm{ReLU}(\mathrm{BN}({\mathrm{Conv}}_{1\times 1}(AF_{t}^{\pm })))\\ E_{s}^{\pm }=\mathrm{ReLU}(\mathrm{BN}({\mathrm{Conv}}_{1\times 1}(AF_{s}^{\pm }))) \end{array}\right.$$

where ReLU(⋅) denotes the ReLU activation and BN(⋅) denotes Batch Normalization. Here, the superscripts + and − indicate manipulation and background regions, respectively.

This process establishes a unified region-level representation basis across teacher and student branches, providing the prerequisite for stable construction of class centers and subsequent prototypical contrastive constraints.

#### 3.3.3. Global Prototype Updating

Region features within a minibatch are sensitive to sample composition and region distribution, which can make class centers unstable. To address this, PCL adopts a two-stage strategy: batch prototype construction followed by global prototype smoothing. Specifically, based on the teacher branch region embeddings obtained in Section 3.3.2, PCL computes class-wise batch prototypes by taking expectations over the batch and spatial dimensions:

(12) $$BP^{\pm }=E_{B,n}[E_{t}^{\pm }]$$

where $BP^{+},BP^{-}$ denote, respectively, the manipulation region prototype and the background region prototype in the current batch, and $E_{B,n}[\cdot ]$ denotes expectation over batch and spatial dimensions. Because single batch statistics may fluctuate, PCL further maintains global manipulation/background prototypes $GP^{\pm }$ with EMA:

(13) $$GP^{\pm }\leftarrow m_{2}\cdot GP^{\pm }+(1-m_{2})\cdot BP^{\pm }$$

where $m_{2}$ is the prototype momentum coefficient controlling the update rate. Compared with batch prototypes, global prototypes accumulate class information over time and provide smoother, more reliable semantic centers.

#### 3.3.4. Intraclass Pulling and Interclass Pushing

PCL aims to pull student manipulation features toward the manipulation prototype and push them away from the background prototype; for background features, the direction is reversed. This explicitly improves intraclass compactness and interclass separability, alleviating feature overlap between manipulation and background under complex degradation. Accordingly, the prototype contrastive losses $L_{PCL}^{\pm }$ for manipulation and background regions are defined as follows:

(14) $$L_{PCL}^{\pm }=-\frac{1}{\left|Y^{\pm }\right|}\sum_{n=1}^{\left|Y^{\pm }\right|}\log\frac{\exp(sim({\tilde{E}}_{s,n}^{\pm },{\tilde{GP}}^{\pm })/\tau )}{\exp(sim({\tilde{E}}_{s,n}^{\pm },{\tilde{GP}}^{+})/\tau )+\exp(sim({\tilde{E}}_{s,n}^{\pm },{\tilde{GP}}^{-})/\tau )}$$

where $\left|Y^{+}|,|Y^{-}\right|$ denote, respectively, the number of valid pixels in the manipulation mask and background mask; sim(⋅,⋅) denotes cosine similarity [62]; ${\tilde{E}}_{s}^{\pm },{\tilde{GP}}^{\pm }$ denote respectively, L2 normalized features and prototypes; ${\tilde{E}}_{s,n}^{\pm }$ denotes the feature at pixel n; and τ is the temperature coefficient. The final PCL loss is as follows:

(15) $$L_{PCL}=L_{PCL}^{+}+L_{PCL}^{-}$$

Through this constraint driven by prototypes, the student branch preserves clearer class boundaries under degradation and achieves more stable manipulation localization.

## 4. Experiments

To comprehensively evaluate ProCoS, experiments were conducted from four perspectives: experimental setup, performance and robustness on SEI-Asym, generalization across datasets and ablation analysis, and computational complexity.

### 4.1. Experimental Setup

This section introduces the SEI-Asym construction process, implementation and training configurations, and evaluation metrics used for unified comparison.

#### 4.1.1. SEI-Asym Dataset

To support manipulation localization research on SEI images under compound degradation, we construct SEI-Asym, a manipulation localization dataset for SEI scenarios. Its design emphasizes both scenario specificity and task difficulty. Instead of reusing generic manipulated objects, SEI-Asym focuses on representative targets centered on key evidence in SEI workflows. Moreover, SEI manipulation localization often involves semantically similar but unmanipulated distractors, so the manipulation localization model must accurately localize truly manipulated regions in the presence of semantically similar distractors.

SEI-Asym is constructed from periodic elevator inspection scenarios. The original images are resized proportionally with the long side set to 512 pixels, and zero padding is applied along the short side to form 512 × 512 inputs. As summarized in Table 2, SEI-Asym contains two manipulation types: FaceSwap and Splice. FaceSwap samples are generated using FaceFusion [47], with segmentation masks produced by SegFace [48]. Splice samples are constructed by using SAM [45] to obtain target objects and masks, followed by synchronized random transformation and pasting into regions with similar semantics and structures. The scaling range is set to 0.72 to 1.0, the rotation range to −5° to 5°, and the perspective perturbation strength to 0.015 to 0.065, with Poisson blending [46] used for fusion. Edge masks are further generated from manipulation masks through a dilation-minus-erosion operation with a kernel size of 5. All samples undergo postprocessing, where slight Gaussian blur with a 3 × 3 kernel is first applied, followed by JPEG compression with a quality factor of 90, to simulate mild degradation during SEI image acquisition and transmission.

**Figure 6:**
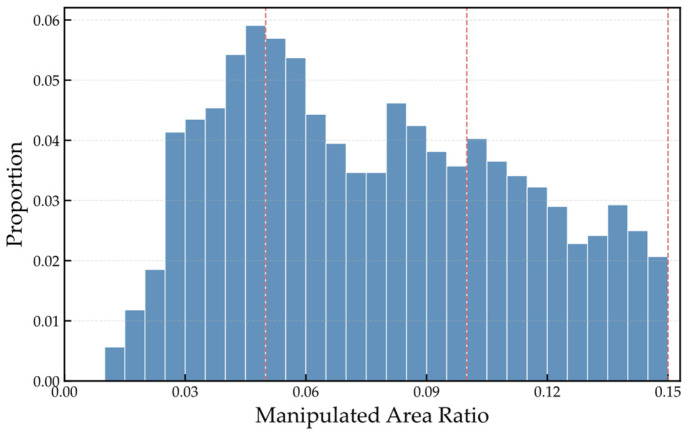
Distribution of manipulated area ratios in SEI-Asym. The histogram reports the sample proportion across different ratios of manipulated areas, showing that samples are mainly concentrated in the controlled range of 0.01 to 0.15 for balanced localization difficulty. The red vertical dashed lines from left to right indicate the threshold values of 0.05, 0.10, and 0.15, respectively.

A manual secondary quality check is then performed to remove samples with unreasonable semantics, obvious blending artifacts, or inaccurate annotations, ensuring both image quality and label reliability. In addition, to avoid unstable evaluation caused by overly small manipulated areas, and to avoid overly easy discrimination caused by overly large manipulated areas, the ratios of manipulated areas are controlled within 0.01–0.15 for all samples, as shown in Figure 6. This range is determined according to the practical object scales of faces and nameplates in SEI images, balancing concealment, learnability, and scale distribution. The final dataset includes 2732 FaceSwap samples and 2987 Splicing samples, split into training and validation sets at a ratio of 8:2. Overall, SEI-Asym is specifically designed for robust manipulation localization in SEI scenarios, from target selection and task difficulty to degradation setting and statistical constraints, and provides the required data foundation for subsequent comparisons and robustness evaluation under compound degradation.

#### 4.1.2. Model Training Configuration

To ensure that the experimental results reflect the performance upper bound of the proposed method while enabling fair comparison with representative methods, we follow a unified and reproducible training and evaluation protocol, as detailed in Table 3. We use SegFormer MiT-B3 as the backbone and initialize it with ADE20K [63] semantic segmentation pretrained weights provided by mmSegmentation [64]. The compared methods are based on the standard implementations provided by IMDLBenCo [13], which systematically reproduces multiple image manipulation detection and localization methods with settings that are identical or close to those in the original papers. Meanwhile, the proposed method is aligned as much as possible with IMDLBenCo in terms of data split, main training procedure, and the hyperparameter settings listed in Table 3.

For generalization evaluation on public benchmarks, we follow the MVSS protocol [16]: training on CASIA v2 and testing on COVERAGE, Columbia, NIST16, CASIA v1, and IMD2020. All training and inference experiments are conducted on a single NVIDIA RTX 3090 GPU workstation.

#### 4.1.3. Evaluation Metrics

To comprehensively evaluate model performance in SEI scenarios, we use metrics from two aspects: localization accuracy and degradation robustness.

For standard localization performance, we adopt F1 score (F1), Area Under the Curve (AUC), and Intersection over Union (IoU) [13]. The pixel-level F1 uses a threshold of 0.5 and jointly reflects precision and recall for detection of manipulated regions. AUC evaluates overall separability between manipulation and background, and IoU measures overlap quality between predicted and ground-truth-manipulated regions.

For robustness under compound degradation, we further introduce a performance drop metric ΔF1 and a stability metric S. ΔF1 measures the loss induced by degradation, defined as the difference between the F1 on clean images $F1_{orig}$ and that on degraded images $F1_{deg}$:

(16) $$\Delta F1=F1_{orig}-F1_{deg}$$

A smaller value indicates that performance degradation is better controlled. S evaluates robustness consistency across different compound degradation combinations and is defined as follows:

(17) $$S=\frac{1}{Std(\Delta F1)}$$

where Std(ΔF1) denotes the standard deviation across all compound degradation conditions; a larger S indicates smoother performance variation across different degradation combinations and thus stronger stability.

### 4.2. Localization Performance and Robustness Evaluation on SEI-Asym

We evaluate ProCoS on SEI-Asym from two aspects: regular localization performance and robustness under degradation. The analysis covers region detection capability, region coverage quality, and stability under both single and compound degradation.

#### 4.2.1. Quantitative Comparison of Localization Performance

To compare the overall localization performance on SEI-Asym, we select representative pixel-level manipulation localization models as baselines, as shown in Table 4. These methods include MVSS-Net [16], ObjectFormer [26], PSCC-Net [29], TruFor [20], IML-ViT [23], and Mesorch [27], covering CNN, Transformer, and hybrid architectures.

ProCoS achieves the best or tied best performance on all three metrics, with F1 = 0.6531, AUC = 0.9670, and IoU = 0.5485. This indicates that the proposed method can effectively distinguish manipulated pixels from background pixels and produce more complete manipulation masks. Compared with the second-best model TruFor, ProCoS improves F1 and IoU by 0.88% and 4.82%, respectively. Although Mesorch reaches the same AUC as ProCoS, ProCoS further improves F1 and IoU by 2.77% and 0.55%, respectively, suggesting that ProCoS can more stably convert discrimination confidence into effective binary localization results.

The differences between the compared methods further reflect the difficulty of SEI-Asym. Methods based on multiscale supervision, multi-cue fusion, or hybrid CNN and Transformer modeling show relatively better results, indicating that both local artifacts and semantic information are useful for SEI manipulation localization. In contrast, ObjectFormer, PSCC-Net, and IML-ViT perform worse in terms of F1 and IoU, suggesting that object-level relations, spatial and channel correlations, or pure global modeling alone are insufficient for small evidence-related objects, same semantic interference, and weak local boundaries in SEI scenarios. Overall, Table 4 shows that ProCoS achieves a more balanced performance in global discrimination, region detection, and mask coverage than representative methods from different technical routes.

#### 4.2.2. Qualitative Comparison of Localization Performance

**Figure 7:**
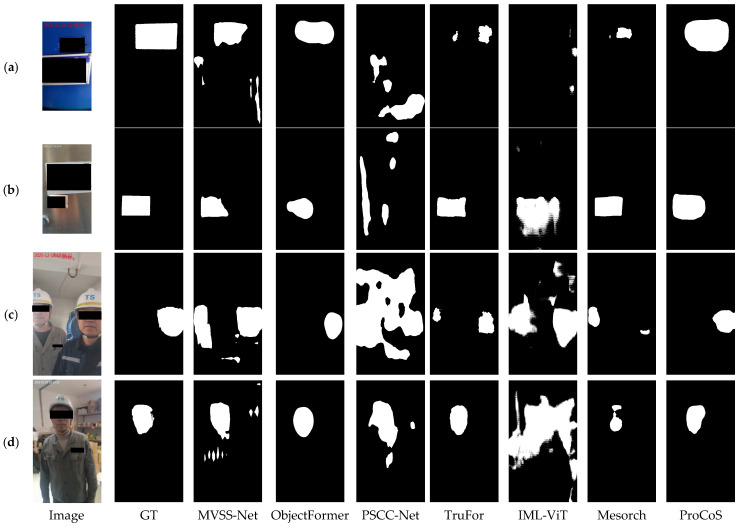
Visual comparison of manipulation localization results on representative SEI-Asym samples. Cases (**a**,**b**) are Splicing, and cases (**c**,**d**) are FaceSwap. The columns show the original image, GT, and the predictions of MVSS-Net [16], ObjectFormer [26], PSCC-Net [29], TruFor [20], IML-ViT [23], and Mesorch [27], and the proposed ProCoS. Black boxes indicate privacy masking only and do not affect localization analysis.

To further evaluate localization performance across two typical SEI manipulation types, we present visual comparisons on four representative samples from SEI-Asym, as shown in Figure 7. The results are consistent with Table 4: ProCoS produces predictions that better match ground-truth-manipulated regions, provides more complete coverage of manipulated targets, and remains focused on truly manipulated objects even when semantically similar background targets exist. In contrast, some baseline models can activate local salient artifacts but often suffer from fragmented masks, insufficient coverage, or increased false positives. Therefore, Figure 7 further confirms that ProCoS provides more stable practical localization in both region completeness and suppression of false positives.

#### 4.2.3. Robustness Evaluation Under Single Degradation Conditions

**Figure 8:**
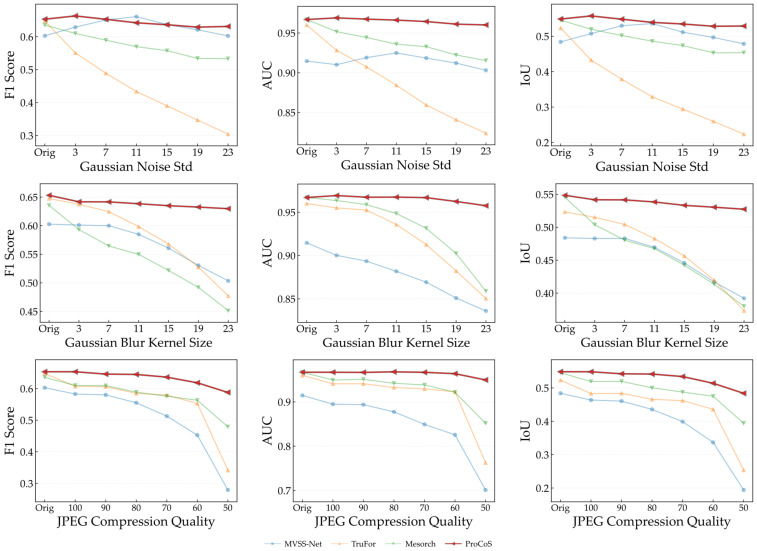
Robustness trends of different methods under three single degradation conditions. The three rows from top to bottom correspond to Gaussian noise, Gaussian blur, and JPEG compression, respectively, while the three columns from left to right show F1, AUC, and IoU, respectively. In each subplot, the horizontal axis denotes the degradation intensity, and the vertical axis denotes the corresponding evaluation metric. Consistent with the metrics in Table 4, this figure shows robustness changes under single degradations from the perspectives of manipulated region detection, overall pixel-level discrimination, and region coverage quality.

Figure 8 reports the robustness results under single degradation settings. We include only models with F1 > 0.5 under clean conditions, namely MVSS-Net [16], TruFor [20], Mesorch [27] and ProCoS. For models that already fail to localize effectively under clean observations, performance fluctuation under degradation may mainly reflect weak baseline localization capability rather than true degradation robustness.

As degradation intensity increases, all methods show performance degradation, but the decline magnitude and stability differ clearly across degradation types. Under Gaussian noise, TruFor shows the most obvious decrease in F1 and IoU as the noise standard deviation increases, indicating its sensitivity to noise perturbation, while Mesorch also presents a continuous degradation trend. In contrast, ProCoS maintains the highest or near highest F1 and IoU in most cases, and its AUC remains in the top range with a smaller decline, suggesting that it can better preserve pixel-level discrimination between manipulated and background regions under noise interference. Under Gaussian blur, ProCoS consistently leads in all three metrics, with a relatively slow decrease in AUC. This indicates that ProCoS not only maintains stable global discrimination, but also preserves better region coverage quality when edge information is weakened. Under JPEG compression, MVSS-Net and TruFor degrade more clearly under strong compression, while Mesorch remains competitive under moderate compression but also declines when the quality factor becomes low. ProCoS achieves the best overall results across the three metrics, especially maintaining higher F1 and IoU under low quality factors. This suggests that the proposed method better suppresses the impact of local detail destruction caused by compression artifacts.

Overall, Figure 8 shows that ProCoS maintains more stable performance under Gaussian noise, Gaussian blur, and JPEG compression, from the perspectives of detection effectiveness, global discrimination, and region coverage quality. These results indicate that the cross-observation consistency and prototype-based separation constraints help the model reduce over reliance on fragile local artifacts and preserve more robust manipulation representations under single degradation conditions.

#### 4.2.4. Robustness Evaluation Under Compound Degradation Conditions

To further examine model robustness under the joint effects of multiple degradations, this work constructs nine compound degradation combinations based on an L9 orthogonal experimental design, and analyzes them from two perspectives: performance degradation for each combination and overall robustness distribution. Under the compound degradation settings of the orthogonal design, validation images are sequentially degraded by Gaussian noise, Gaussian blur, and JPEG compression. Each degradation type is assigned three intensity levels, namely high, medium, and low, corresponding to noise standard deviations of 7, 15, and 23, blur kernel sizes of 7, 15, and 23, and JPEG quality factors of 90, 70, and 50, respectively.

**Figure 9:**
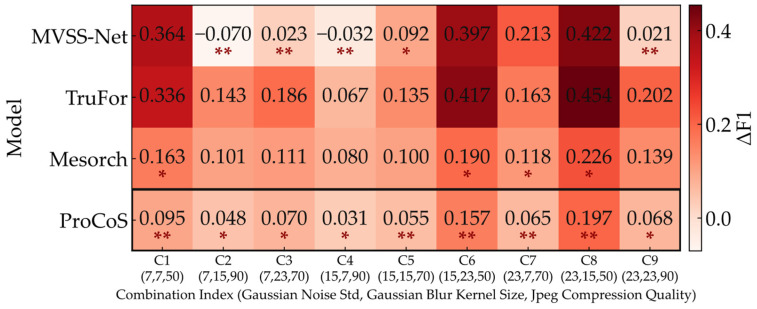
Performance degradation matrices of different methods under compound degradation conditions, across nine orthogonal compound combinations. The x-axis is the combination index; the values in parentheses indicate the three degradation levels; each cell value is ΔF1 under that combination; a darker color indicates a larger drop induced by degradation; “**” denotes the best in that column; and “*” denotes the second-best.

Figure 9 presents the performance degradation matrix of different methods under each compound degradation combination. As shown in Figure 9, different methods exhibit clear differences under compound degradations. MVSS-Net maintains certain stability under some mild or moderate degradation combinations, indicating that multiscale local supervision can provide a degree of buffering against degradation perturbations, but this advantage is not sustained across most combinations. TruFor shows strong global discrimination under regular conditions, but suffers relatively clear performance drops when multiple degradations act jointly, suggesting that its multi-source cues may still be affected by the superposition of noise, blur, and compression artifacts. Mesorch remains competitive in several combinations, indicating that its hybrid architecture has certain adaptability to complex degradations. In contrast, ProCoS achieves the smallest performance degradation in most columns among the nine compound degradation combinations. In particular, it obtains the best results within the corresponding columns under the more severe compound degradation combinations C5, C6, and C8, indicating that the proposed method can more effectively maintain stable discrimination between manipulated and background regions when local textures, edge details, and compression traces are simultaneously damaged.

**Figure 10:**
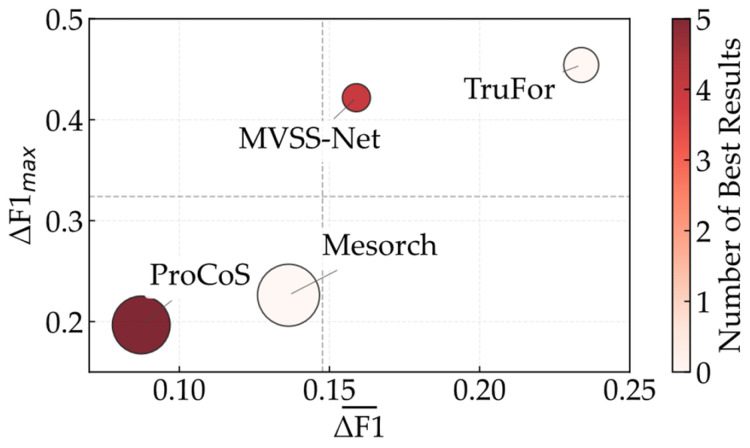
The comprehensive distribution of robustness for different methods under compound degradation conditions. It summarizes robustness from a statistical perspective. The x-axis is the average ΔF1 across the nine combinations, i.e., $\bar{\Delta F1}$; the y-axis is the maximum ΔF1, i.e., ${\left(\Delta F1\right)}_{\max}$; color indicates the number of best column results; and bubble size is determined by the stability metric S.

Figure 10 further summarizes the overall performance of different methods under all compound degradation conditions from a statistical perspective, including the average performance degradation $\bar{\Delta F1}$, maximum performance degradation ${\left(\Delta F1\right)}_{\max}$, number of best results, and stability index S. As shown in Figure 10, ProCoS achieves $\bar{\Delta F1}$ = 0.0873 and ${\left(\Delta F1\right)}_{\max}$ = 0.1966, both outperforming the compared methods. This indicates that the proposed method not only suffers lower performance loss under most degradation combinations but also better controls the degradation magnitude in the worst case. In other words, the robustness advantage of ProCoS does not come from occasional superiority under individual combinations, but is reflected simultaneously in average performance, extreme degradation control, and cross-combination stability.

Although MVSS-Net remains competitive under some compound degradation combinations, its $\bar{\Delta F1}$ and ${\left(\Delta F1\right)}_{\max}$ are still significantly higher than those of ProCoS, indicating that its overall robustness remains limited when multiple degradations are superimposed. Mesorch shows strong stability, but its average performance degradation is still higher than that of ProCoS, suggesting that its stability may not be fully translated into stronger degradation resistance. Overall, ProCoS exhibits strong interference resistance under compound degradation scenarios.

### 4.3. Generalization Across Datasets and Ablation Experiments

This section further analyzes model generalization across datasets and the contribution of key modules through ablation.

#### 4.3.1. Generalization Across Datasets

Generalization across datasets is reported in Table 5 using F1 and AUC on public benchmarks. As shown in Table 5, the advantage of ProCoS is not limited to a single dataset. Instead, it remains competitive across diverse distributions. On five public datasets, ProCoS achieves the best F1 and AUC simultaneously on Columbia, CASIA v1, and IMD2020. On COVERAGE and NIST16, although ProCoS does not obtain the highest F1, it still achieves the best AUC values (0.9340 and 0.8668), indicating that ProCoS maintains more stable pixel-level separability, although this advantage is not fully reflected in the final F1.

From the global statistics, ProCoS obtains an average F1 of 0.5691 and an average AUC of 0.9324, where average AUC is the best among all compared models. Even after excluding the abnormal NIST16 case of IML-ViT, ProCoS still maintains the best overall average performance. These results indicate that ProCoS’s advantage across datasets does not come from overfitting to a single public dataset, but from more stable modeling of relationships between manipulation and background across different test domains.

#### 4.3.2. Ablation Experiments

Ablation results are shown in Table 6, including F1 under clean conditions, average F1 under three single degradation conditions, $\bar{\Delta F1},{\left(\Delta F1\right)}_{\max}$ under compound degradation, and the stability metric S. Five experiment groups are constructed progressively: Group 1 uses only the SegFormer MiT-B3 backbone; Group 2 adds the ConvNeXt local detail path and gated fusion module; Group 3 further adds an Edge Head with edge supervision; Group 4 introduces DRSL to improve robustness across observations; and Group 5 adds PCL to form the full ProCoS, using dual prototypes for manipulation and background to further optimize feature space structure.

Table 6 shows that module contributions are not simple linear accumulation but a progressive pattern of localization enhancement, robustness degradation, robustness recovery, and stability strengthening. Group 1 yields an F1 of 0.4808, indicating limited baseline localization ability. After adding local detail modeling and edge supervision (Groups 2 and 3), F1 improves by 24.50% and 31.10% over Group 1, respectively, but robustness deteriorates clearly. Although these groups achieve relatively high S, their $\bar{\Delta F1}$ and ${\left(\Delta F1\right)}_{\max}$ indicate that robustness is not truly improved.

After introducing DRSL (Group 4), F1 further increases slightly, while $\bar{\Delta F1}$ decreases by 68.75%, ${\left(\Delta F1\right)}_{\max}$ drops to 0.2351, and S is also significantly improved compared with Group 1. This shows that DRSL improves localization while restoring stable discrimination under degraded observations.

With PCL added (Group 5), F1 slightly adjusts to 0.6531, but average F1 under all three single degradation conditions becomes the best among the five groups. Meanwhile, $\bar{\Delta F1}$ and ${\left(\Delta F1\right)}_{\max}$ are further reduced, and stability is further improved. This indicates that PCL stabilizes class structure between manipulation and background and further suppresses both fluctuation and performance degradation in the worst case under compound degradation.

Overall, ProCoS improves localization via local detail modeling, restores robustness across observations via DRSL, and further strengthens stable class separation via PCL, achieving a better balance between accuracy and robustness.

### 4.4. Computational Complexity Analysis

To evaluate practical computational cost, we compare different models in terms of parameter count and Floating Point Operations (FLOPs), as reported in Table 7. Here, parameters reflect model size, and FLOPs indicate theoretical inference cost. Since ProCoS keeps only the student branch during inference, we additionally report the complexity of ProCoS (w/o Teacher) to distinguish training cost from deployment cost. Because some baseline implementations use different default input resolutions, complexity statistics follow each model’s standard configuration.

The results show that although the full ProCoS has a relatively large parameter count, its FLOPs are still lower than those of MVSS-Net, PSCC-Net, TruFor, and IML-ViT. After removing the teacher branch, ProCoS is reduced to 75.169M parameters and 67.456G FLOPs, approximately half of the full model and close to ObjectFormer. Combined with the performance, robustness, and generalization results above, this indicates that the gains of ProCoS are not achieved at unacceptable extra cost, but rather under controllable inference complexity with strong localization accuracy and degradation robustness.

## 5. Conclusions

We investigate manipulation localization for SEI images. Targeting the challenges specific to SEI, severe degradation interference, and limited support from existing datasets/evaluation settings, we propose ProCoS, construct SEI-Asym, and establish a compound degradation evaluation protocol based on orthogonal experimental design. Extensive experiments support the following conclusions.

1. To address unstable cross-observation responses and localization drift under compound degradation, we introduce DRSL, which explicitly incorporates consistency between clean and degraded observations into training. The results show that DRSL improves standard localization performance while significantly reducing performance collapse induced by degradation under compound degradation. Therefore, for pixel-level forensic tasks under degraded conditions, explicitly modeling cross-observation consistency is a key route to improving robustness.
2. To mitigate feature overlap between manipulation and background regions under compound degradation, we further introduce PCL. Experimental results show that PCL further improves robustness under both single and compound degradation settings. Therefore, contrastive constraints based on prototypes can effectively suppress performance fluctuation induced by degradation, and this insight is potentially transferable to other visual localization tasks sensitive to degradation.
3. To fill the gap in data foundations for SEI and realistic robustness evaluation, we build SEI-Asym and the compound degradation protocol based on orthogonal design. Under this setting, ProCoS achieves an F1 of 0.6531, an AUC of 0.9670, and an IoU of 0.5485. Therefore, for industrial visual forensics and trustworthy inspection, dataset construction, evaluation protocol design, and model innovation should be developed in a unified manner for SEI scenarios.

Although this study demonstrates strong performance, robustness, and generalization, limitations remain. SEI-Asym currently focuses on FaceSwap and Splicing, and broader manipulation types should be included in future work. In addition, the current compound degradation setting can be further extended to better reflect the randomness and temporal variability of real pipelines for acquisition, transmission, and processing. Future research will continue toward more realistic degradation modeling and broader trustworthy visual analysis focused on SEI.

## Figures and Tables

**Table 1:** Comparison of the existing image manipulation localization datasets with SEI-Asym.

Main Scenario	Dataset	Manipulation Type	Designed for SEI Scenarios	Considers Same Semantic Interference
General local manipulation	CASIA v1/v2 [49]	Splice, Copy-Move	×	×
	Columbia [50]	Splice	×	×
	Coverage [51]	Copy-Move	×	×
	NIST16 [52]	Splice, Copy-Move, Removal	×	×
	IMD2020 [53]	Splice, Copy-Move, Removal	×	×
Face forgery	FaceForensics++ [54]	FaceSwap, etc.	×	×
	OpenForensics [55]	FaceSwap, etc.	×	×
	ForgeryNet [56]	FaceSwap, etc.	×	×
SEI	SEI-Asym	FaceSwap, Splice	√	√

In the columns “Designed for SEI Scenarios” and “Considers Same Semantic Interference”, “√” indicates that the method satisfies the corresponding criterion, while “×” indicates that it does not.

**Table 2:** Composition and design highlights of the SEI-Asym dataset.

Type	Object	Generation Method	Annotation	Postprocessing	Count
FaceSwap	Inspector face	(1) FaceFusion [47] generates face-swapped results; (2) SegFace [48] generates the segmentation masks.	Pixel-level segmentation masks and edge masks	Mild JPEG compression and Gaussian blur	2732
Splicing	Equipment nameplate	(1) SAM [45] obtains the target object and mask; (2) Splicing is performed after random transformations; (3) Poisson blending [46] is used to smooth boundaries.			2987

**Table 3:** Implementation details and training configuration.

Item	Setting	Item	Setting
Optimizer	AdamW	Batch size	8
Weight decay	0.01	Epochs	100
Initial learning rate	6 × 10^−5^	Input size	512 × 512
Learning rate schedule	Poly	$\;m_{1},m_{2}$	0.999, 0.999

**Table 4:** Quantitative comparison of localization performance of different models on SEI-Asym.

Model	F1	AUC	IoU
MVSS-Net (TPAMI22) [16]	0.6027	0.9147	0.4840
ObjectFormer (CVPR22) [26]	0.4745	0.8952	0.3674
PSCC-Net (TCSVT22) [29]	0.3379	0.7894	0.2265
TruFor (CVPR23) [20]	0.6474	0.9600	0.5233
IML-ViT (Arxiv24) [23]	0.4305	0.8341	0.3136
Mesorch (AAAI25) [27]	0.6355	**0.9670**	0.5455
ProCoS (ours)	**0.6531**	**0.9670**	**0.5485**

The best performance in each column is highlighted in bold.

**Table 5:** Generalization results of different models. The Average column summarizes overall performance across datasets.

Model	COVERAGE		Columbia		NIST16		CASIA v1		IMD2020		Average	
	F1	AUC	F1	AUC	F1	AUC	F1	AUC	F1	AUC	F1	AUC
MVSS-Net [16]	0.2913	0.7339	0.4033	0.7579	0.2407	0.7441	0.5037	0.8484	0.3082	0.7850	0.3494	0.7739
ObjectFormer [26]	0.2966	0.7450	0.3413	0.5325	0.1777	0.7259	0.4292	0.8763	0.1734	0.6810	0.2836	0.7121
PSCC-Net [29]	0.2429	0.6866	0.6029	0.8138	0.2018	0.7300	0.3751	0.8331	0.2589	0.8031	0.3363	0.7733
TruFor [20]	**0.4291**	0.9071	0.8580	0.9250	**0.3479**	0.8331	0.7244	0.9462	0.4342	0.8904	0.5587	0.9004
IML-ViT * [23]	0.4113	0.8639	0.7488	0.8947	-	-	0.7184	0.9398	0.4502	0.8061	**0.5822**	0.8761
Mesorch [27]	0.2366	0.8316	0.5207	0.8310	0.3369	0.8452	0.7209	0.9599	0.3289	0.8587	0.4288	0.8653
ProCoS	0.4078	**0.9340**	**0.8740**	**0.9568**	0.3075	**0.8668**	**0.7909**	**0.9771**	**0.4652**	**0.9274**	0.5691	**0.9324**

* IML-ViT shows abnormally low localization results on NIST16 due to a padding issue; therefore, its Average is computed over the other four datasets only for fair comparison. The best performance in each column is highlighted in bold.

**Table 6:** Ablation study of major components and learning mechanisms in ProCoS.

Group	SegFormer	ConvNeXt + Gated Fusion	Edge Head	DRSL	PCL	F1	Avg0. F1 (JPEG)	Avg0. F1 (Blur)	Avg0. F1 (Noise)	$\bar{\Delta F1}$	${\left(\Delta F1\right)}_{\max}$	S
1	√	-	-	-	-	0.4808	0.3735	0.5767	0.4331	0.1524	0.2719	9.26
2	√	√	-	-	-	0.5986	0.4638	0.4365	0.5625	0.4791	0.5349	24.55
3	√	√	√	-	-	0.6303	0.5298	0.5595	0.5934	0.3607	0.3862	**34.34**
4	√	√	√	√	-	**0.6630**	0.6192	0.6256	0.6217	0.1127	0.2351	17.37
5	√	√	√	√	√	0.6531	**0.6309**	**0.6365**	**0.6622**	**0.0873**	**0.1966**	19.45

“√” indicates that the corresponding module is included in the model configuration, whereas “-” indicates that the module is not included. The best performance in each column is highlighted in bold.

**Table 7:** Comparison of model complexity.

Model	Input Size	Parameters (M)	FLOPs (G)
MVSS-Net [16]	512 × 512	150.528	171.008
ObjectFormer [26]	224 × 224	134.144	65.197
PSCC-Net [29]	256 × 256	3.668	376.832
TruFor [20]	512 × 512	68.697	236.544
IML-ViT [23]	1024 × 1024	91.778	590.848
Mesorch [27]	512 × 512	85.754	124.928
ProCoS	512 × 512	150.338	149.504
ProCoS (w/o Teacher)	512 × 512	75.169	67.456

## Data Availability

The raw data supporting the conclusions of this article will be made available by the authors upon request.

## Abbreviations

The following abbreviations are used in this manuscript:

SEI	Special Equipment Inspection
ProCoS	Prototypical Contrastive-Synergistic Network
DRSL	Degradation-Resilient Synergistic Learning
PCL	Prototypical Contrastive Learning
ECA	Entropy-modulated Consistency Alignment
DCT	Discrete Cosine Transform
EMA	Exponential Moving Average
GT	Ground Truth
KL	Kullback–Leibler
F1	F1 Score
AUC	Area Under the Curve
IoU	Intersection over Union
FLOPs	Floating Point Operations
